# Code Red: Explaining Average Age of Death in the City of Hamilton

**DOI:** 10.3934/publichealth.2015.4.730

**Published:** 2015-11-17

**Authors:** Patrick F. DeLuca, Pavlos S. Kanaroglou

**Affiliations:** Centre for Spatial Analysis, School of Geography and Earth Sciences, McMaster University, 1280 Main Street West, Hamilton, Ontario, Canada, L8S 4K1

**Keywords:** code red, spatial analysis, factor analysis, Hamilton, poverty

## Abstract

The aim of this study is to identify the underlying factors that explain the average age of death in the City of Hamilton, Ontario, Canada, as identified in the Code Red Series of articles that were published in the city's local newspaper in 2010. Using a combination of data from the Canadian Census, the Government of Ontario and the Canadian Institute for Health Information, factor analysis was performed yielding three factors relating to poverty, working class, and health and aging. In a regression analysis these factors account for 42% of the total variability in the average ages of death observed at the census tract level of geography within the city.

## 1. Introduction

The determinants of health approach [1], is an important part of health research and health policy in both Canada and overseas (for example, [2]). The Canadian Public Health Agency has created a twelve item list of what determines health that include both individual and neighbourhood determinants [3]. Included in this list are biology and genetic endowment, gender, culture, healthy child development, health services, social support networks, personal health practices and coping skills, physical and social environments, income and social status, education and literacy, and employment conditions. Implicit in some of these items is the issue of inequality.

Politicians, the media and the academic literature point to the problem of income inequality and the effects that it has on various facets of life. Specifically with regards to public health, the link to income inequality has been well researched [4]–[9]. Many of these studies however are at a global or regional scale of geography [10], [11]. Inequalities also have health impacts at much smaller levels of geography such as within a city [12], with the city of Hamilton, Ontario, Canada being an excellent example. Past research into health issues in Hamilton are numerous and are often linked with inequality [13]–[19].

In April of 2010 the issue of inequality and health was brought to the public's attention through a seven day series of articles published in Hamilton's local newspaper, The Hamilton Spectator [20]. The series, entitled “Code Red” consisted of twenty-four mapped variables, interviews with stakeholders, simple statistics and relevant photographs interwoven into a coherent story about the health of the city, told in a way that could easily be comprehended by The Spectator's lay audience. It successfully highlighted such disparities as a 21 year difference in average age of death, an 89 fold difference in secondary school dropout rates, a six-fold difference in dwelling values and other stark differences between the most and least prosperous neighbourhoods of the city.

The reaction to Code Red was generally positive and had numerous effects. It galvanized public opinion, became a major issue in the subsequent mayoral election, and prompted the City to create a Neighbourhood Development Office. It also was a factor in the location of two university health care facilities, and it inspired changes to secondary school curricula and lead to donations for scholarships to be allocated to worthy students from the poorest parts of the city [21].

Code Red evidently had (and continues to have) a profound effect on the community. In general, it has been attributed to the overall approach employed in publishing the series. One of the great strengths of the study is that metrics provided were very simple for both policy makers and a lay audience to understand. Additionally, the visualization through mapping added a sense of place to the study, which people were able to identify with. It is important to note, however, that this study was descriptive in nature, with none of the reported disparities statistically tested.

The aim of this research is to examine one of the most striking disparities discussed in the Code Red series, that being the variation seen in the average age of death in the city of Hamilton. This variation is examined using the remaining twenty-three variables (plus a few others derived from the 2006 Census), thereby including all of the main components to the initial Code Red Study.

## 2. Study Area

**Figure 1.**
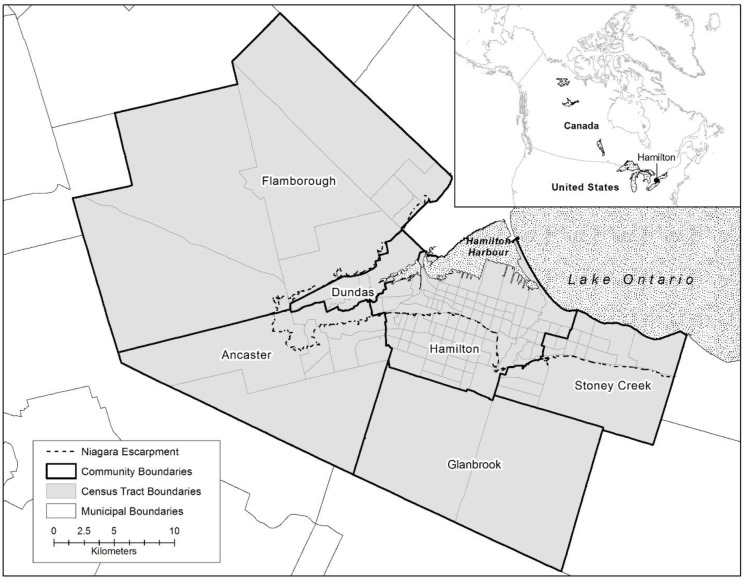
The City of Hamilton.

The City of Hamilton, Ontario (Figure 1) is a midsize industrial Canadian city located on the shores of Lake Ontario (43.3°N, 79.9°W). The city, as it is constituted today, is the result of an amalgamation (that took place in 2001), of five generally affluent suburban communities (Ancaster, Dundas, Flamborough, Glanbrook, and Stoney Creek) and a central urban area (the old City of Hamilton), geographically divided into the upper and lower city by the Niagara Escarpment (Figure 1). At the time of the initial Code Red study, the population of the city (n = 505,000) had people from all parts of the socio-economic spectrum, and has been recognized as having a significant proportion of families and individuals living at or beneath Canada's poverty level. Specifically, the lower city has lower average dwelling values, lower incomes, highest number of children living in poverty, and the greatest percentage of government transfers (Table 1). Over the past 50 years, as industrial jobs have been slowly leaving the city, wealthier people moved out of older neighbourhoods in the lower city, into the newer, less dense suburban communities, and immigrants and individuals of lower socio-economic status (SES) moved into lower-cost housing in the inner city.

**Table 1.** Selected socio-economic variables for the amalgamated City of Hamilton^a^

Communities	Average Dwelling Value	Median Family Income	% Children below low income cut-off	Government Transfers as % of total income
Former City of Hamilton	$197 819	$58 283	24.0	16.2
Lower City	$176 981	$52 118	28.7	18.0
Upper City	$227 979	$67 206	17.3	13.6
Stoney Creek	$272 447	$76 377	11.6	11.6
Glanbrook	$305 495	$81 432	4.1	10.2
Ancaster	$396 976	$103 651	3.5	5.8
Dundas	$305 856	$79 350	12.3	8.8
Flamborough	$367 121	$91 305	3.6	6.8

^a^Source: 2006 Canadian Census, average of census tract values

## 3. Methods

Data for the study was obtained from several sources. Death records along with the postal code of residence at time of death (N = 12,115) were obtained from Service Ontario for 2006–2008 and were used to compute the average age of death per census tract. Several variables relating to social determinants of health were collected from the 2006 Census of the Canadian population. Variables collected include dwelling value, percentage of female lone parent, median income per person over age 15, median income per family, people over 15 living below the poverty line, children living below the poverty line, people aged 25–64 with no High School Diploma, people aged 25–64 with a University Degree, percentage of immigrants, percentage of people aged 65 and older, percentage of dwellings occupied by renters, percentage of people living alone and percentage of people either single, divorced or widowed. From the census data, the GINI Coefficient for income inequality was derived. Emergency room visit data and hospital admission records for the period of April 1, 2006–March 31, 2008 were collected from Canadian Institute for Health Information (CIHI) for every patient listing Hamilton as a home address (approximately 400,000 observations). Each health record included age, gender of patient, the date of emergency room or hospital visit, the length of stay, the medical (ICD-10) coding for every procedure performed and a geographic marker (dissemination area of residence). Variables that were derived from the data obtained from CIHI include: average length of stay for acute hospital care per hospital admission, average number of days of alternate level care per hospital admission, hospital admission rate per 1,000 people, urgent hospital admission rate per 1,000 people, rate of respiratory related emergency room visits per 1,000 people, rate of cardiovascular related emergency room visits per 1,000 people, percentage of emergency room visits with patients reporting no family physician, emergency room visit rate for trauma for children under age 16 per 1,000 people, emergency room visit rate for trauma for persons aged 70 and over per 1,000 people, total emergency room visit rate per 1,000 people, and rate of psychiatric related emergency room visits per 1,000 people. All of the aforementioned records did not include any personal information that would allow the identities of individual patients to be revealed. Both local District School Boards (Catholic and Public) provided data on high school completion by dissemination area, from which the dropout rate per 1,000 students was computed. All observations were either geocoded (in the case of postal codes) or aggregated (in the case of dissemination areas) to the census tract level of geography used by Statistics Canada to measure a wide variety of social and economic variables within urban regions of Canada. They have the added benefit in this study of having boundaries that match up well with the City's traditional neighbourhood boundaries in the urban core. In total, there are 135 census tracts in the amalgamated City of Hamilton but due to data suppression, small population sizes or missing data, 6 census tracts were omitted from subsequent analysis.

Initially, a matrix was constructed to explore correlations among the variables used in the analysis. To further characterise the census tracts, a combination of factor analysis, a global measure of spatial autocorrelation and local indicators of spatial association (LISA) were employed. Factor analysis was employed as a data reduction technique (deemed necessary due to the large number of variables and the potential for spurious relationships) to identify the number of latent constructs and the underlying factor structure of the set of variables, while accounting for the common variance in the data set [22]. All variables except average age of death were included in this analysis which employed a varimax rotation to preserve orthogonality amongst the resulting factors. A scree test, the proportion of variance explained and communality scores were used to determine the final number of factors to retain for subsequent analysis. For a variable to remain in the analysis, a communality score of at least 0.6 was employed. A choropleth map of each factor along with a measure of global spatial autocorrelation and a LISA map were created to visualize the results and aid in the interpretation of the factors. Specifically, global Moran's I was used to assess the degree of clustering that exists in the average ages of death across the city of Hamilton. Local Moran's I statistic was used to identify spatial clusters or subareas of high or low average ages of death, in addition they were used examine the different factors from the factor analysis. These statistics identify the association between a single unit of analysis (in this case a census tract) and its neighbours based on distance or adjacency [23]. In this case, a nearest-neighbour approach was employed based on rook weights (i.e., sharing a common boundary), and the resulting contiguity matrix was row-standardized. These statistics are particularly well-suited to: (1) identify the existence of clusters; (2) assess the assumptions of stationarity; and (3) determine the distance beyond which no discernible spatial association exists [23]. Finally, each factor was then entered into an ordinary least squares regression model with average age of death as the dependent variable.

## 4. Results

**Figure 2.**
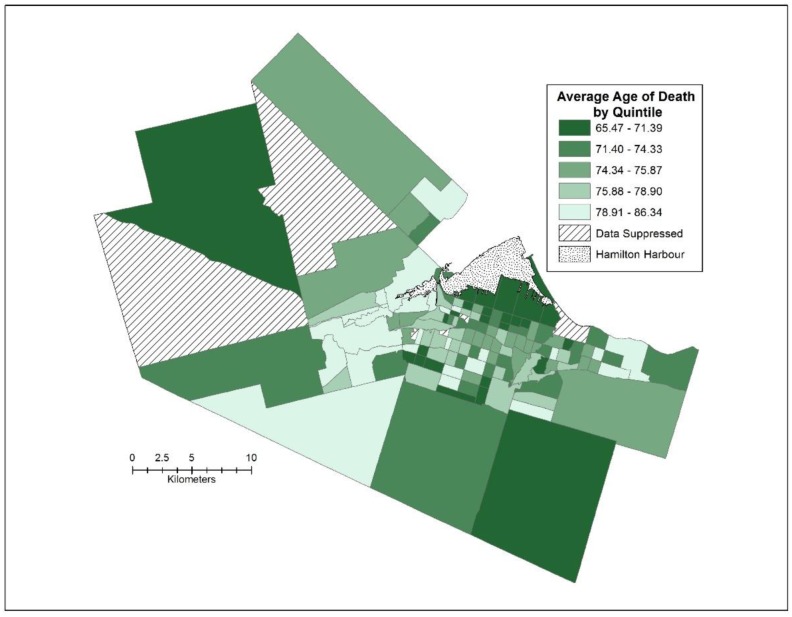
Average of death by quintile, City of Hamilton, 2006–2008.

**Figure 3.**
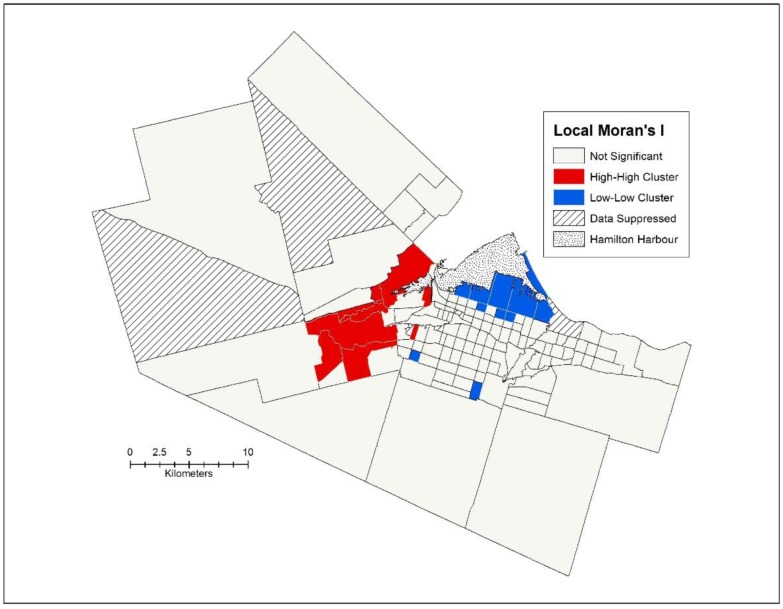
Local Moran's I statistic applied to average of death, 2006–2008.

The average age of death in Hamilton from 2006–2008 was 75.09 years for males and females combined. When examined by census tract, the range was 20.87 years with the lowest average age of death for a census tract being 65.47, and the highest being 86.34. As Buist [20] pointed out, the neighbourhood with the lowest average of death would be ranked 165^th^ in the world, on par with Nepal, while the census tract with the highest average of death was five years better than Canada's ranking for life expectancy. Spatially, the pattern is clear, 17 of the 26 census tracts in the lowest quintile of average age of death are in the lower city of Hamilton, with a further 7 census tracts in the upper city (Figure 2). Most of the highest average ages of death are in Ancaster and Dundas. From a global perspective, there is statistically significant spatial autocorrelation in the average ages of death, with a Moran's I score of 0.274 (*p* < 0.001). This pattern is further confirmed through the Local Moran's I statistic (figure 3), clearly depicting the Low-Low cluster (i.e., a census tract with a low average age of death surrounded by other census tracts with low average ages of death) in the industrial North-End, bordering the harbour, while the High-High cluster (i.e., a census tract with a high average age of death surrounded by other census tracts with high average ages of death) can be seen in most of Dundas and the urban parts of Ancaster.

Twenty-four variables were entered into the factor analysis. Of these, seventeen were retained and formed three factors based on the scree test, the proportion of variance explained, and the communality scores. The decision to remove the other seven variables from the analysis was based on communality scores less than 0.6, or if the variable was an ultra-Heywood case (as was the case with the Emergency Room visits per 1000 variable). Table 2 shows the seventeen variables, how they load onto each of the factors, the communality score and the proportion of variance explained by each factor.

**Figure 4.**
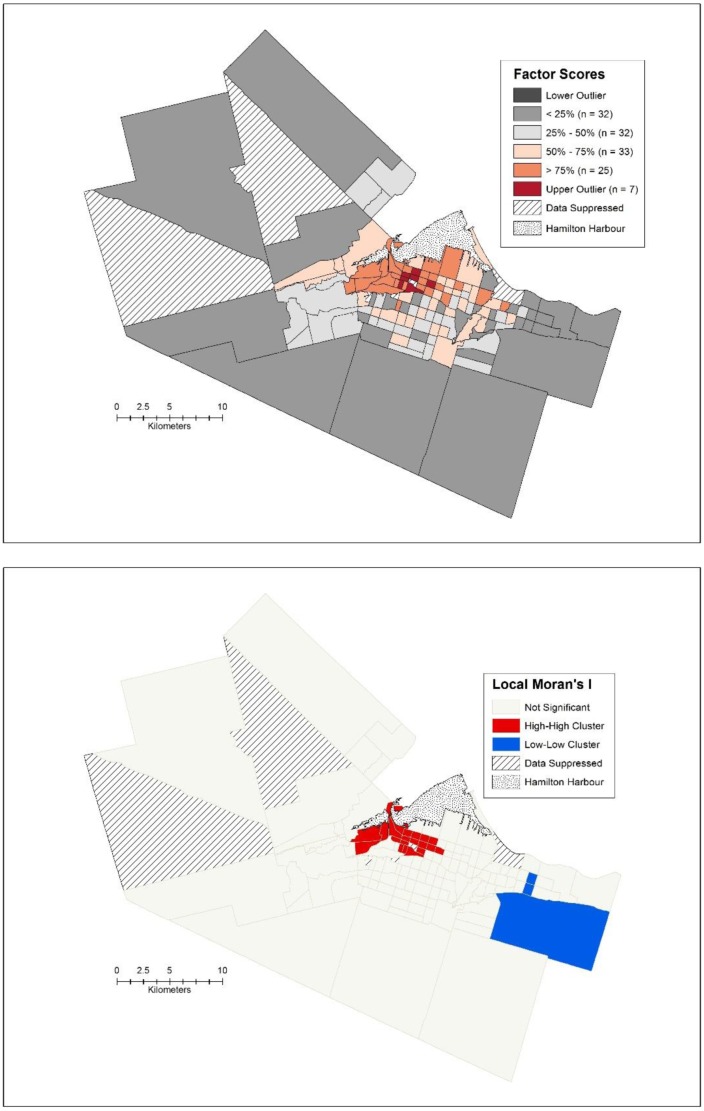
a) Spatial box plot of factor scores and b) Local Moran's I for the poverty factor.

The first factor is related to poverty, and has very high loadings for percent low income cut off, percent renters, percent of people who do not have a family doctor, rate of psychiatric admissions per 1000, percent of people living alone and the Gini coefficient. Spatially, the pattern of the factor scores is clustered as displayed through the spatial box plot, Local Moran's I (figure 4a and 4b) and global Moran's I (I = 0.689, *p* < 0.001). The highest factor scores are in the western part of the urban core in the lower city of Hamilton, with a cluster of seven outliers in the downtown core itself. Generally, the more affluent suburbs can be easily seen in figure 4a as they all have low factor scores for this particular factor, with three census tracts constituting a significant cluster of low factor scores (figure 4b).

**Figure 5.**
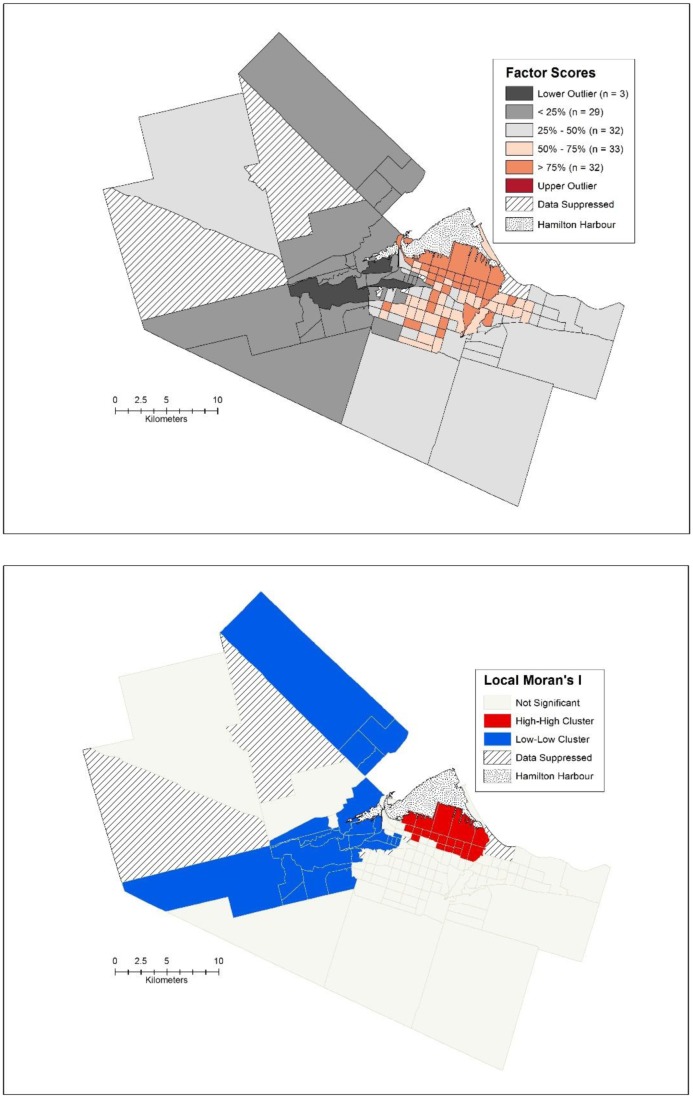
a) Spatial box plot of factor scores and b) Local Moran's I for the low-income, working class factor

Factor two is related to the low-income, working class of the city. It is characterized by lower dwelling values, lower median family incomes, higher numbers of female lone parent families and a lack of post-secondary education. Spatially, the pattern exhibits a greater degree of clustering than the poverty factor (Moran's I = 0.795, *p* < 0.001) and is shifted east-ward in the urban core of the lower city (Figure 5a). The census tracts in this area are close to the industrial and manufacturing areas of the city that abut the harbour. On the opposite end of the spectrum, the lower factor scores are in the suburbs, with statistically significant clustering occurring in the more affluent areas of Ancaster, Dundas and parts of Flamborough (Figure 5b)

**Figure 6.**
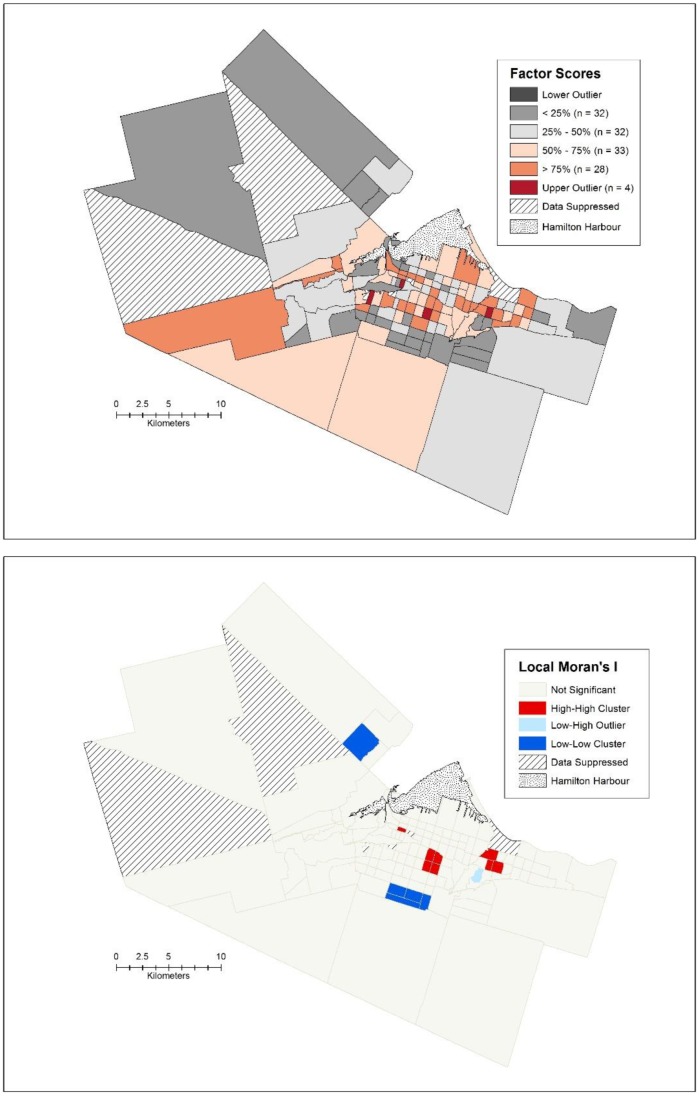
a) Spatial box plot of factor scores and b) Local Moran's I for the health and aging factor.

Factor three is related to health and aging, and is characterized by census tracts with high percentages of people over age 65, higher admission to hospital rates per 1000, higher urgent admissions per 1000, higher cardiovascular disease admissions per 1000, and higher percentage of people who are single, divorced or widowed. The spatial pattern is not as dominant for this factor as it is for the other two (figure 6a), with the highest factor scores scattered throughout the city of Hamilton and suburbs. As such, the spatial clustering, while present in this factor, is considerably less than the other two factors (Moran's I = 0.278, *p* < 0.001). Figure 6b reveals both high and low clusters for the factor scores, with clusters of high factor scores being located in the east-end of the urban core, bordering with an older part of Stoney Creek and another one in the older part of the upper city. Clusters of low factor scores are located in the part of Flamborough known as Waterdown and at the southern end of the upper city of Hamilton. Both of these areas are similar in that at the time of the census when the data was collected they were new subdivisions dominated by younger families.

The three factors were entered into an ordinary least squares regression model with average age of death as the dependent variable (Table 3). From this model it is evident that as the factors related to poverty and low income/working class increase, the average age of death decreases. As the health and aging factor increases, so too does the average age of death. In total, this model, which is statistically significant (F = 31.31, *p* < 0.001) explains about 42% of the total variability of the average age of death in Hamilton. The residuals were tested for normality (with the Jarque-Bera = 0.05, *p* = 0.98), spatial autocorrelation (Moran's I = 0.02, *p* = 0.59), and heteroskedasticity (Koenker = 6.46, *p* = 0.10), with each test showing that the model is valid.

**Table 2.** Factor Analysis Results

Variable	Factor 1 Poverty	Factor 2 Working Class	Factor 3 Health & Aging	Communality
Dwelling Value	-0.401	-0.748	-0.185	0.755
Female Lone Parent Family	0.586	0.622		0.730
Median Family Income	-0.600	-0.673	-0.256	0.878
Percent Under Low Income Cut Off	0.878	0.408		0.937
Percent No Post-Secondary Education	0.309	0.820	0.145	0.789
Percent Post-Secondary Education	0.195	-0.888	-0.157	0.851
Percent Secondary School Dropout	0.592	0.516		0.617
Percent Age Greater than 65	-0.112	-0.165	0.828	0.725
Percent Rent	0.819	0.152	0.290	0.778
Hospital Admission Rate per 1000	0.302	0.254	0.761	0.735
Urgent Admission Rate per 1000	0.346	0.198	0.841	0.866
Cardiovascular Disease Admissions per 1000		0.258	0.852	0.792
Percent of People without a Family Doctor	0.837			0.701
Rate of Psychiatric Admissions per 1000	0.787	0.240	0.222	0.726
Percent Single, Divorced, Widowed	0.458	0.378	0.677	0.811
Percent of People Living Alone	0.711		0.516	0.772
Gini Coefficient	0.799	0.411	0.373	0.946
Proportion of Variance Explained	0.335	0.229	0.227	
Cumulative Variance Explained	0.335	0.564	0.791	

**Table 3.** Regression results of factors against average of death

Coefficient	Beta	Standard Error	t	p-value
Intercept	75.122	0.287	261.741	0.000
Poverty Factor	-0.739	0.292	-2.533	0.013
Low income/Working Class Factor	-2.261	0.297	-7.604	0.000
Health and Aging Factor	1.618	0.295	5.497	0.000

Adjusted R^2^ = 0.415

## 5. Discussion

When Code Red was first published in April of 2010, it had the sub-title of “Where You Live Affects Your Health” and it was a deliberate choice of the authors to not include any complicated statistical analysis that would be beyond the grasp of The Hamilton Spectator's readership [21]. As has been evidenced, Code Red had (and continues to have) a profound effect on the community, and this was attributed to the overall approach employed in publishing the series. It is relatively common that health risks are misrepresented by the media [24], [25], often overemphasizing the roles of the health care system and personal health habits, while underemphasizing the role of social determinants [26]. Such was not the case here as the Code Red project was a partnership between the journalist and academics, as opposed to the usual approach where the journalist depends on published research to generate a story. Further, while the health care system was discussed, it received equal coverage to the role of social determinants. However, while the series' descriptive nature was effective, without any statistical evidence to support the narrative, it was difficult to measure how much of an impact any of the included variables had on health. This research fills that void, with the goal of examining how much of an effect the collection of ‘Code Red’ variables had on the average age of death.

Factor analysis was employed to identify the number of latent constructs and the underlying factor structure of the set of variables used in the initial Code Red study. In addition to this, the Gini coefficient was included to address more directly the issue of inequality. While not used in the original Code Red study, it was employed in two follow-up studies related to teenage pregnancies and cancer. Other variables added to the analysis were related to social deprivation, namely, the percent of people living alone and the percent of people single, divorced or widowed [27]. The first factor, which was related to poverty had many self-explanatory variables including people living under the low income cut-off, percent of people who rent their home rather than own it and income inequality scores for the census tract of residence. Mental health, as measured by the rate of psychiatric admissions per 1000, has been shown to be related to poverty [28], [29]. People with mental illness face many barriers over their lifetime which may prevent them from securing adequate education and employment. The lack of employment, in turn, affects the ability to earn an adequate income. As a result, people with mental illness may eventually drift into poverty [30]. It has also been well-established in the literature that low-income families often have barriers to accessing primary health care [31], [32], making the percent of people without a family doctor a logical inclusion in the poverty factor as well. The spatial pattern of this factor is well known in Hamilton with the downtown core of the city having high factor scores for poverty. This area of the city has also been identified as an area of concern in [13], [15], [19] and [33].

The second factor, related to low-income working class includes dwelling value, median family income, percent lone female parents and a lack of post-secondary education. The key difference between the first and second factor is that the latter is characterized by home ownership rather than renting and the lack of education loads much higher in this second factor than in the first. Spatially, this pattern has shifted eastward matching findings from [34], who highlighted (albeit with data from an earlier census) that Northeast Hamilton had higher percentages of people with low educational attainment and high percentages of lone parent families, along with lower average dwelling values and incomes when compared to the rest of the City of Hamilton.

The third factor of health and aging included census tracts with high percentages of people over age 65, higher admission to hospital rates per 1000, higher urgent admissions per 1000, higher cardiovascular disease admissions per 1000, and higher percentage of people who are single, divorced or widowed. The variable capturing senior citizens aside, the remaining variables which had high loadings in this factor are all expected to be higher amongst seniors than younger age groups. Spatially the absence of clusters as large as those seen in the other two factors would be expected as one wouldn't expect seniors to cluster in several adjacent census tracts within the city.

When these three factors were combined in a regression model they were able to account for a statistically significant amount of the variation present in the average of death, in total accounting for approximately 42 percent of the total variation. The single biggest factor according to the regression coefficients in this model was the low-income, working class, followed by health and aging, with poverty coming in third, still explaining a statistically significant amount of variation. This association between poverty and health is substantial and the effects can result in lifelong body burden, starting from childhood. In comparison to other Canadian cities, the degree of poverty in Hamilton is higher, and the poor are more segregated, clustering in the center of the city [35], but as to whether the same factors explain as much of the variation in the average of death in other cities still needs to be tested statistically.

Mitigation of the effects of poverty requires a public health approach, with interventions to increase economic opportunity, investments in early childhood development, nutrition (including breakfast programs), education and healthy communities. One of the most important outcomes of Code Red was the establishment of a neighbourhood action strategy to help address the issues raised in the series. Based solely on the initial descriptive analysis and maps, the main elements of this action strategy are the establishment of resident-led neighbourhood plans, an increased investment into the poorer neighbourhoods and the building of new partnerships to support healthy neighbourhoods [36]. While this analysis did not inform any of the initial policy discussion, it will be made available for future discussions around the social determinants of health in the city. With regards to the ‘next steps’ in the policy discussion around health and poverty, it is the intention of the authors of the original series to create a follow up series, examining what impact the initial series and the neighbourhood action plans have had on the overall health of the City of Hamilton. An analysis similar to what was conducted here will follow to determine if poverty and low income/working class still plays such an important role in explaining the overall observed pattern in the average age of death across the City.
